# Combining manipulation of transcription factors and overexpression of the target genes to enhance lignocellulolytic enzyme production in *Penicillium oxalicum*

**DOI:** 10.1186/s13068-017-0783-3

**Published:** 2017-04-20

**Authors:** Liwei Gao, Zhonghai Li, Chengqiang Xia, Yinbo Qu, Meng Liu, Piao Yang, Lele Yu, Xin Song

**Affiliations:** 1 State Key Laboratory of Microbial Technology, School of Life Science, Shandong University, Jinan, 250100 Shandong China; 2 National Glycoengineering Research Center, Shandong University, Shan Da Nan Road 27, Jinan, Shandong 250100 China; 3 Department of Bioengineering, Qilu University of Technology, Jinan, 250353 Shandong China

**Keywords:** *Penicillium oxalicum*, Transcription factor, Cellulase, Hemicellulase

## Abstract

**Background:**

Lignocellulolytic enzymes are the main enzymes to saccharify lignocellulose from renewable plant biomass in the bio-based economy. The production of these enzymes is transcriptionally regulated by multiple transcription factors. We previously engineered *Penicillium oxalicum* for improved cellulase production via manipulation of three genes in the cellulase expression regulatory network. However, the potential of combinational engineering of multiple regulators and their targets at protein abundance and activity levels has not been fully explored.

**Results:**

Here, we verified that a point mutation XlnR^A871V^ in transcription factor XlnR enhanced the expression of lignocellulolytic enzymes, particularly hemicellulases, in *P. oxalicum*. Then, overexpression of XlnR^A871V^ with a constitutive *PDE_02864* promoter was combined with the overexpression of cellulase transcriptional activator ClrB and deletion of carbon catabolite repressor CreA. The resulted strain RE-7 showed 8.9- and 51.5-fold increased production of cellulase and xylanase relative to the starting strain M12, respectively. Further overexpression of two major cellulase genes *cbh1*-*2* and *eg1* enabled an additional 13.0% improvement of cellulase production. In addition, XlnR^A871V^ led to decreased production of β-glucosidase and amylase, which could be attributed to the reduced transcription of corresponding enzyme-encoding genes.

**Conclusions:**

The results illustrated that combinational manipulation of the involved transcription factors and their target genes was a viable strategy for efficient production of lignocellulolytic enzymes in filamentous fungi. The striking negative effect of XlnR^A871V^ mutation on amylase production was also highlighted.

**Electronic supplementary material:**

The online version of this article (doi:10.1186/s13068-017-0783-3) contains supplementary material, which is available to authorized users.

## Background

Lignocellulosic biomass is the most abundant renewable and sustainable resource on earth and has been regarded as a resource of fermentable sugars for the production of liquid biofuels and biochemicals for a long time [1]. As the main agents of plant decay in the environment, filamentous fungi such as *Trichoderma reesei* [2, 3], *Aspergillus* spp. [4, 5], and *Neurospora crassa* [6, 7] can express a complex mixture of enzymes to synergistically deconstruct the polysaccharides in plant cell walls. Commercial biofuel production remains hindered by the high cost of enzyme production for biomass conversion [8]. Thus, construction of lignocellulolytic enzyme high-producing strains is important to improve the economy of bioconversion of lignocellulosic materials.

*Penicillium oxalicum* wild-type strain 114-2 has been studied for cellulase production for more than 30 years in China [9]. Three main cellobiohydrolases (CBHs), fifteen endoglucanases (EGs), eleven β-glucosidases (BGLs), and fifty-one hemicellulases were predicted to be encoded in its genome [9, 10]. Owing to these enzymes, the lignocellulolytic enzyme system of *P. oxalicum* is more diverse than that of the main industrial strain *T. reesei* [2]. A *P. oxalicum* mutant JU-A10-T, which has enhanced cellulase expression after multiple rounds of mutagenesis and screening, has been applied for commercial cellulase production for 20 years [11]. Comparative genomic analysis of JU-A10-T and 114-2 and subsequent functional verification indicated that a frameshift mutation in the gene encoding carbon catabolite repressor CreA in JU-A10-T strain was responsible for its cellulase hyper-production [11]. The similar case was reported in *T. reesei*, where the truncation of CRE1 (ortholog of CreA) also dramatically increased cellulase expression in high-producing strain RUT-C30 [12]. The function of CreA/CRE1 in regulating various biological processes has been described in many fungi [13–15]. Besides, ClrB/CLR-2, as a pathway-specific transcription factor, is essential for cellulase expression in both *N. crassa* and *Aspergillus nidulans* [16]. In our previous work, a transcription factor gene deletion library was constructed in *P. oxalicum* and several regulators including ClrB, CreA, XlnR, and AmyR were proved to regulate cellulase expression [10]. A strategy of genetically modifying these regulators through gene overexpression and deletion was used to efficiently improve the production of lignocellulolytic enzymes [17]. However, the potential of this strategy has not been fully explored regarding the manipulation of transcription factors at the activity level.

Hemicellulose, as the second most abundant component of lignocellulosic biomass, is a group of heterogeneous polysaccharides including xylan and mannan. [1]. The structural heterogeneity and complex constituents made them require a complex set of enzymes for efficient degradation. The removal of hemicellulose could make cellulose more accessible to cellulolytic enzymes. Thus, improving the production of hemicellulase is a potential strategy for more efficient deconstruction of lignocellulosic biomass [18]. As the major transcriptional activator of xylan degradation and xylose utilization in filamentous fungi, XlnR (orthologs named XYR1 or XLR-1 in different species) has different roles in the regulation of cellulose degradation [19]. In *Aspergillus niger* and *T. reesei*, XlnR/XYR1 was proved to be essential for the expression of both cellulase and hemicellulase genes [20, 21]. However, XLR-1 did not play a significant role in cellulase gene expression in *N. crassa* [18]. In *P. oxalicum*, we also found that XlnR was indispensible for hemicellulase induction and to a less extent for cellulase expression [10]. A point mutation in XYR1 (A824V) in *T. reesei* was responsible for the strong deregulation (i.e., inducer-independent high expression) of both cellulase and xylanase gene expressions [22]. The homologous mutation on XLR-1 in *N. crassa* also resulted in constitutive xylanase expression [22, 23]. These findings offer a potential target for us to further engineer the regulatory pathway for high production of lignocellulolytic enzymes in *P. oxalicum*.

In this study, a point mutation A871V in XlnR was verified to increase the expression of lignocellulolytic enzyme genes in *P. oxalicum*. Furthermore, this mutation was combined with the manipulation of genes encoding CreA and ClrB as well as two main cellulases to evaluate the strategy for improving lignocellulolytic enzyme productions through engineering both regulatory proteins and their targets.

## Results

### Enhanced expression of lignocellulolytic enzyme genes due to the point mutation XlnR^A871V^

**Fig. 1:**
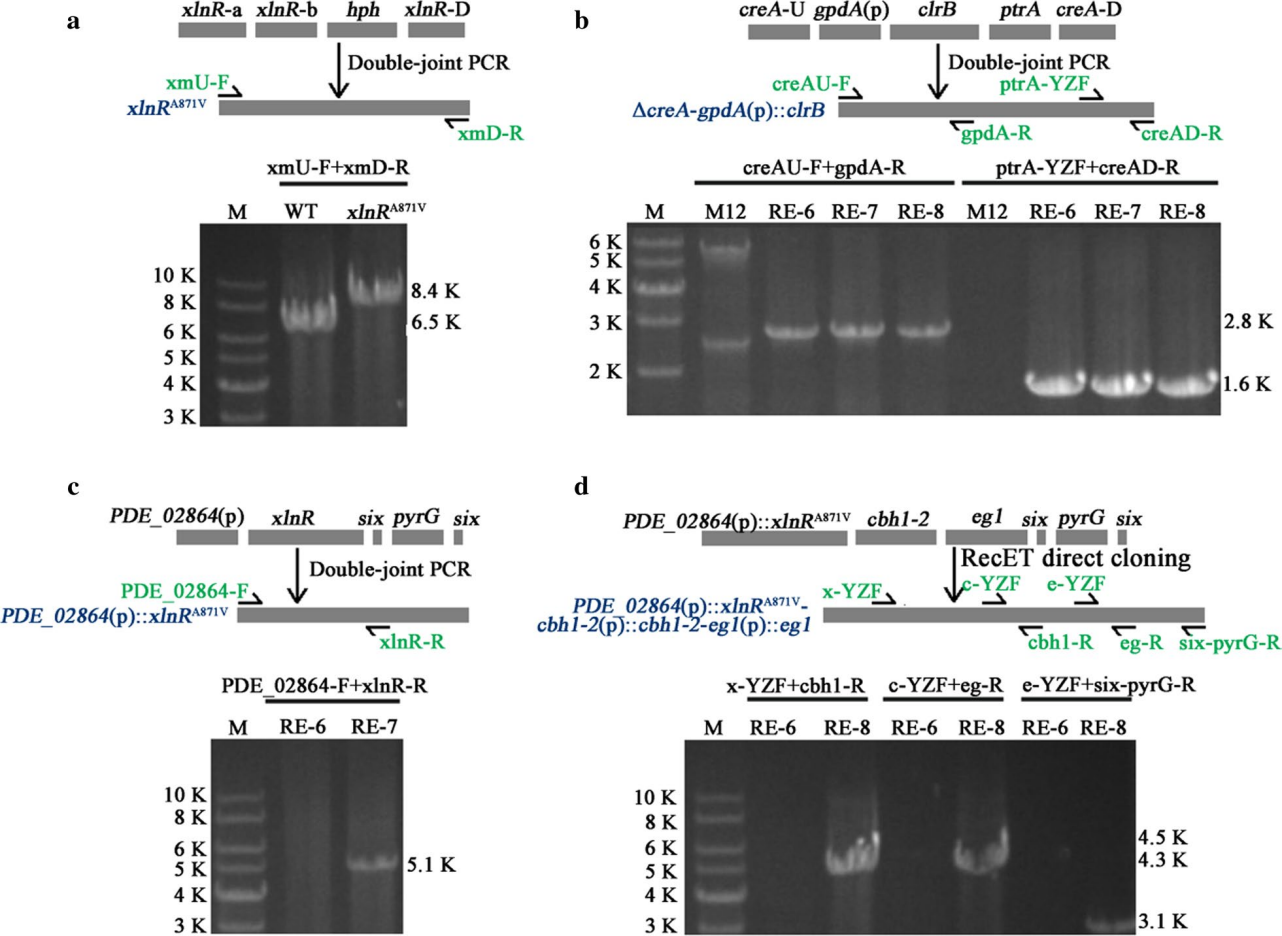
Schematic diagram of the construction of *xlnR* ^A871V^, RE-6, RE-7, and RE-8 mutants. **a** Construction of the *xlnR* ^A871V^ cassette and identification of *xlnR* ^A871V^. Replacement of *xlnR* by *xlnR* ^A871V^ expression cassette resulted in an increase from 6.5 kb in the wild-type strain to 8.4 kb in *xlnR* ^A871V^ strain with primer pair xmU-F + xmD-R. **b** Construction of Δ*creA*-*gpdA*(p)::*clrB* cassette and identification of RE-6. The 2.8 and 1.6 kb fragments were detected in the RE-6, RE-7, and RE-8 strains with primer pairs creAU-F + gpdA-R and ptrA-YZF + creAD-R. **c** Construction of *PDE_02864*(p)::*xlnR* ^A871V^ cassette and identification of RE-7. The 5.1 kb fragment was detected in RE-7 stain with primer pair PDE_02864-F + xlnR-R. **d** Construction of *PDE_02864*(p)::*xlnR* ^A871V^-*cbh1*-*2*(p)::*cbh1*-*2*-*eg1*(p)::*eg1*-*pyrG* cassette and identification of RE-8. The 4.5, 4.3, and 3.1 kb fragments were detected in RE-8 stain with x-YZF + cbh1-R, c-YZF + eg1-R, and e-YZF + six-pyrG-R primer pairs, respectively

In our previous study, we identified that overexpression of *xlnR* resulted in significantly improved xylanase production and a slight increase in cellulase production in *P. oxalicum* [10]. Considering the above-mentioned mutation of XYR1/XLR-1 that markedly enhanced the production of cellulase or xylanase in *T. reesei* and *N. crassa* [22, 23], we examined whether the homologous point mutation XlnR^A871V^ in *P. oxalicum* had the similar effect. Consequently, an *xlnR* ^A871V^ cassette under the control of its native promoter and terminator was constructed (Fig. 1a) and transformed into *P. oxalicum* wild-type strain 114-2. Several stable transformants of *xlnR* ^A871V^ (Table 1) were obtained after two rounds of conidial separation and purification. One of these transformants was identified by PCR amplification (Fig. 1a), Southern blot (Additional file 1A), and DNA sequencing. These results indicated that the *xlnR* ^A871V^ cassette was solely integrated at the native *xlnR* locus in this strain. Neither the promoter and terminator regions nor the coding region of *xlnR* bores any other mutations.

**Table 1:** Strains used in this study

Strain name	Genotype or description	Parent strain	References
114-2	Wild-type	–	[9]
M12	*pyrG* ^Q226*^	114-2	[26]
*xlnR* ^A871V^	*xlnR* ^A871V^-*hph*	114-2	This study
*gpdA*(p)::*xlnR*	*gpdA*(p)::*xlnR*	114-2	[10]
RE-6	*pyrG* ^Q226*^; Δ*creA*-*gpdA*(p)::*clrB*-*ptrA*	M12	This study
RE-7	Δ*creA*-*gpdA*(p)::*clrB*;*PDE_02864*(p)::*xlnR* ^A871V^-*pyrG*	RE-6	This study
RE-8	Δ*creA*-*gpdA*(p)::*clrB*;*PDE_02864*(p)::*xlnR* ^A871V^-*cbh1*-*2*(p)::*cbh1*-*2*-*eg1*(p)::*eg1*-*pyrG*	RE-6	This study

**Fig. 2:**
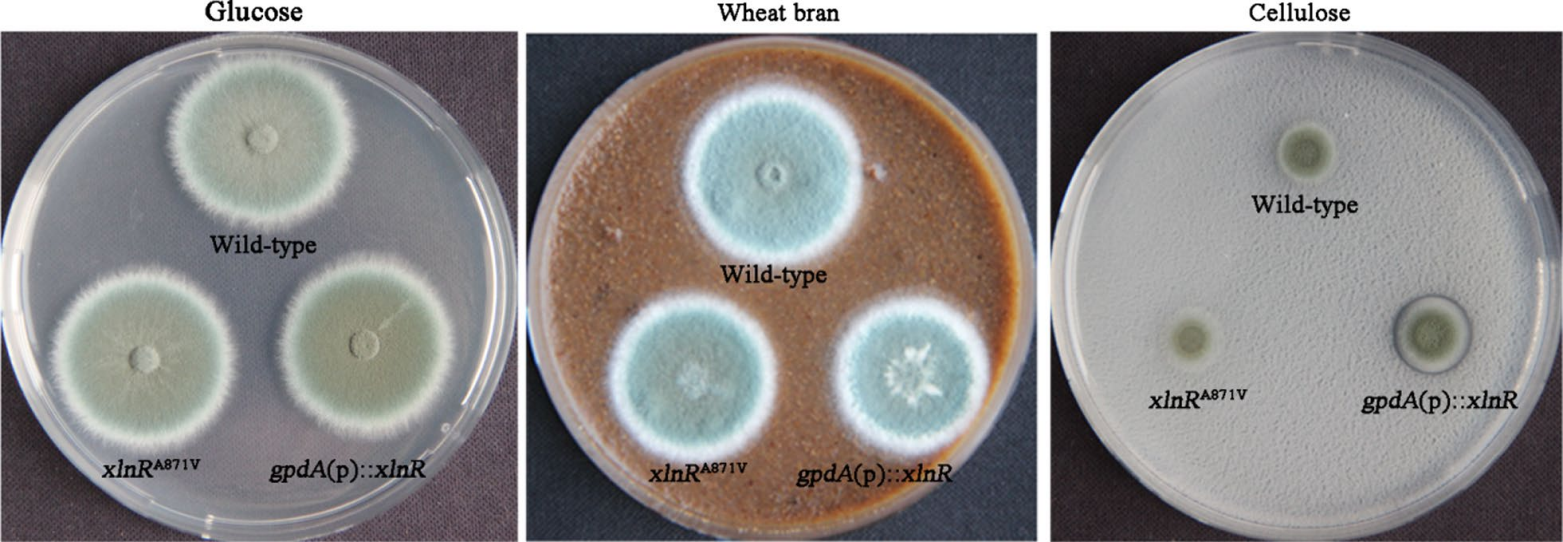
Colony morphology of wild-type, *xlnR* ^A871V^, and *gpdA*(p)::*xlnR* strains on plates containing different carbon sources. Phenotypes of the strains were observed after 4-day cultivation on glucose, wheat bran, and cellulose plates

**Fig. 3:**
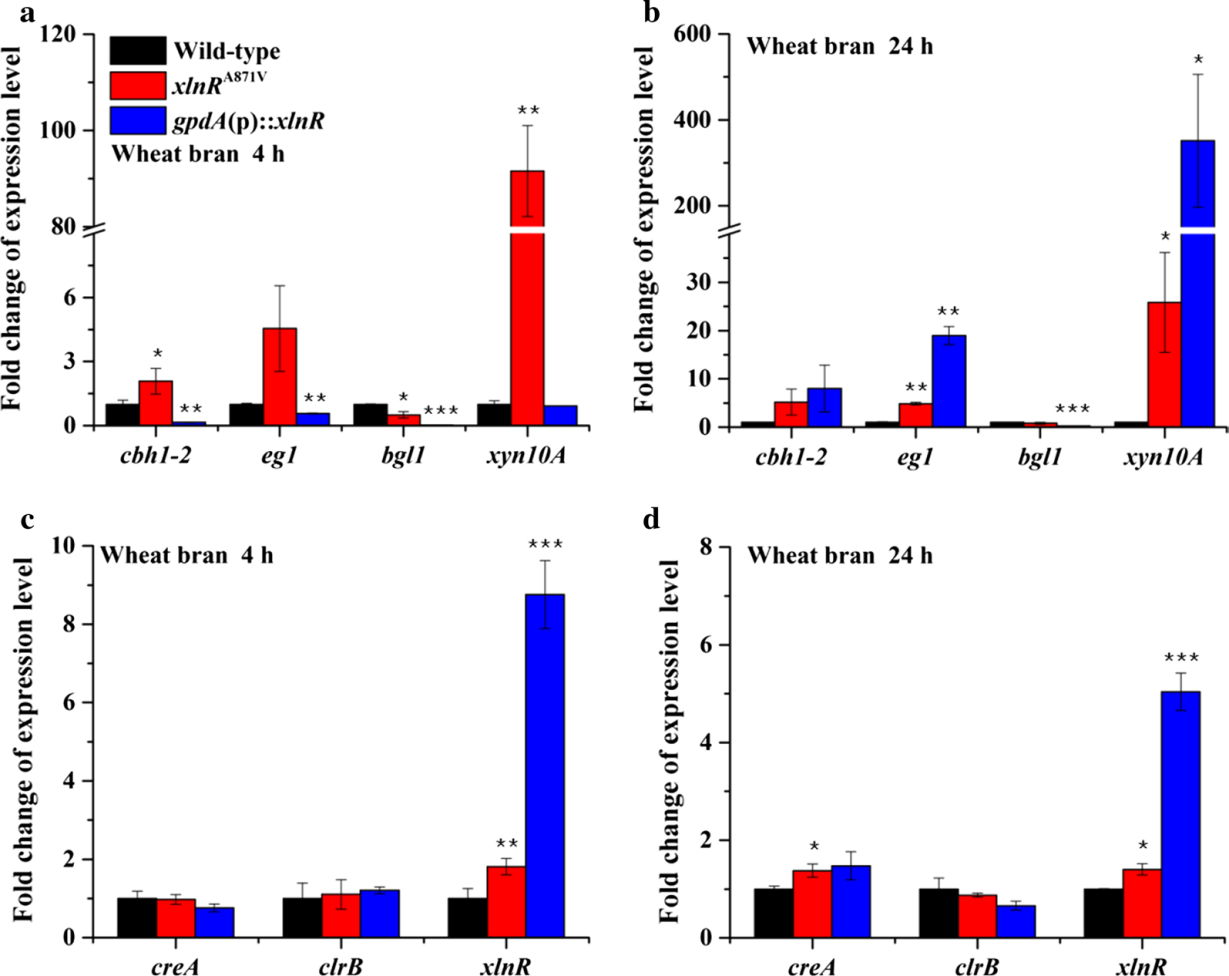
Transcription analysis of lignocellulolytic enzymes and transcription factors in wild-type, *xlnR* ^A871V^, and *gpdA*(p)::*xlnR* strains. Transcriptional levels of lignocellulolytic enzymes (**a**, **b**) and transcription factors (**c**, **d**) in *xlnR* ^A871V^ and *gpdA*(p)::*xlnR* versus those in wild-type strain (set to one) in wheat bran medium at 4 and 24 h were analyzed. *Error bars* represent the standard deviations. Statistical significance of the differences between wild-type and each mutant was calculated. *p < 0.05, **p < 0.01, ***p < 0.001

Given the previously reported enhanced production of xylanase and cellulase in *gpdA*(p)::*xlnR* mutant where *xlnR* was expressed under the control of the *A. nidulans gpdA* promoter [10], we compared the enzyme production abilities of *xlnR* ^A871V^ mutant with those in the wild-type strain and *gpdA*(p)::*xlnR* mutant. The *xlnR* ^A871V^ mutant displayed nearly identical phenotypes on glucose and wheat bran plates to wild-type strain, while *gpdA*(p)::*xlnR* mutant displayed an obvious hydrolysis halo on cellulose (Fig. 2). To examine whether the transcriptional levels of lignocellulolytic genes in *P. oxalicum* respond to XlnR^A871V^, the strains were transferred to wheat bran medium containing xylan and cellulose as inducers. The transcription of major xylanase gene *xyn10A* in the *xlnR* ^A871V^ mutant showed a 91.6-fold increase at 4 h and 24.8-fold increase at 24 h compared to those in the wild-type strain (Fig. 3a, b). Transcriptional levels of major cellulase genes *cbh1*-*2* and *eg1* also increased by 1.1 to 4.2-folds (Fig. 3a, b). In contrast, the transcription of major β-glucosidase gene *bgl1* [24, 25] showed decrease in both *xlnR* ^A871V^ and *gpdA*(p)::*xlnR* than those in wild-type strain (Fig. 3a, b). Neither the overexpression nor the mutation of *xlnR* affected the transcription of *creA* and *clrB* (Fig. 3c, d). The above results demonstrated that the mutation XlnR^A871V^ facilitated the expression of xylanolytic enzymes, and moderately the expression of cellulolytic enzymes, in *P. oxalicum*.

**Fig. 4:**
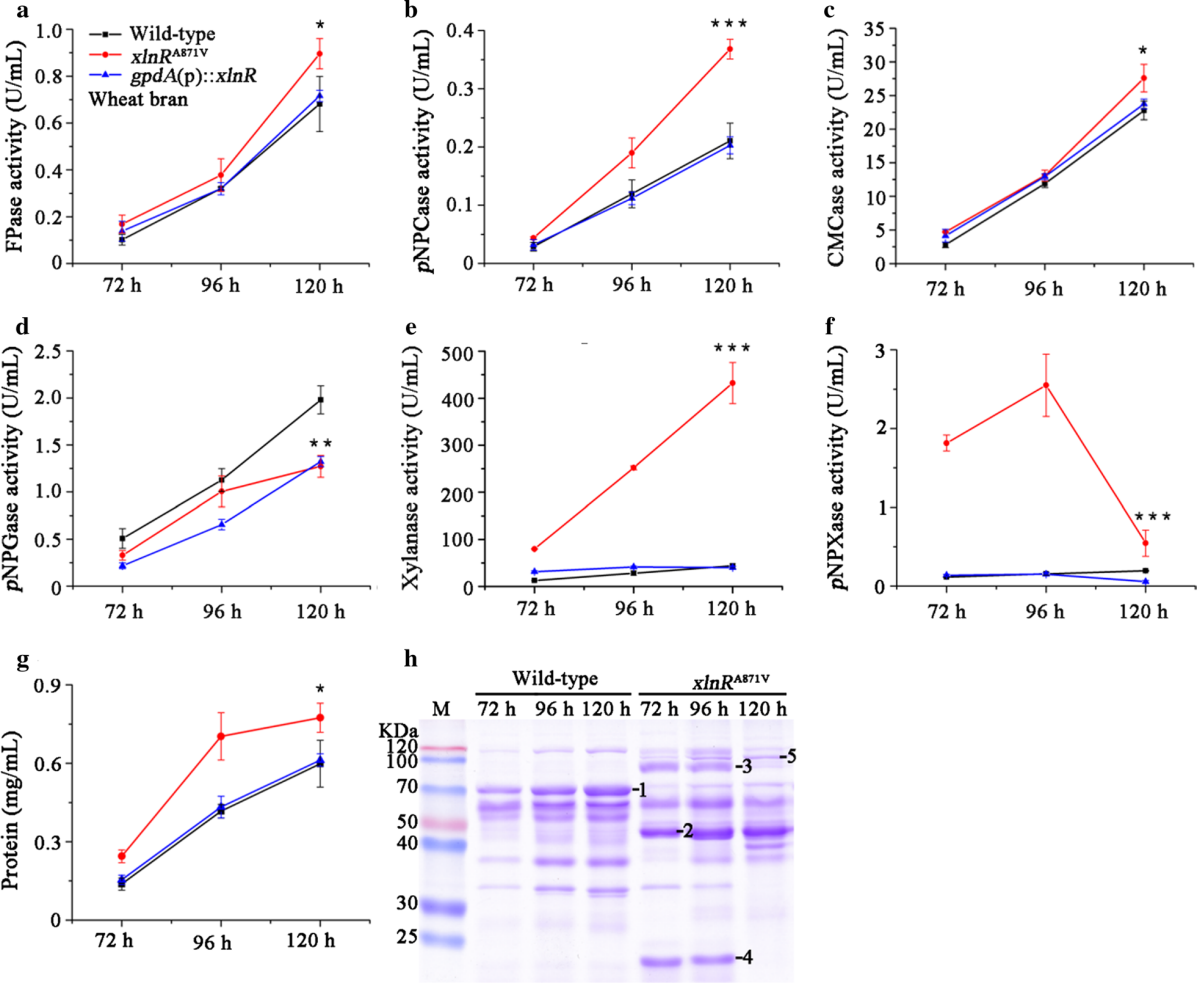
Effect of the point mutation XlnR^A871V^ on the production of extracellular enzymes on wheat bran. FPase (**a**), *p*NPCase (**b**), CMCase (**c**), *p*NPGase (**d**), xylanase (**e**) and *p*NPXase activities (**f**), and extracellular protein concentrations (**g**) of supernatants from wild-type, *xlnR* ^A871V^, and *gpdA*(p)::*xlnR* strains were analyzed. All *error bars* represent the standard deviations. Statistical significance of the differences between wild-type and *xlnR* ^A871V^ at 120 h was calculated. *p < 0.05, **p < 0.01, ***p < 0.001. **h** SDS-PAGE analysis of the supernatants of the wild-type and *xlnR* ^A871V^ strains. The protein bands 1–5 were identified by MS–MS as listed in Table 2

To investigate whether the enzyme production levels correspond to the change of transcriptional levels in *xlnR* ^A871V^ mutant, various lignocellulolytic enzyme activities were analyzed when the strains were grown in wheat bran medium. The *xlnR* ^A871V^ mutant showed significant increases in FPase (filter paper enzyme), *p*NPCase (CBH), CMCase (EG), xylanase, *p*NPXase (β-xylosidase) activities, and extracellular protein concentration compared to wild-type strain after 120-h cultivation (Fig. 4a–c, e–g). Particularly, the production of xylanase increased by 8.9-fold in *xlnR* ^A871V^ (Fig. 4e). On the other hand, the level of *p*NPGase (BGL) was significantly reduced in the mutant (Fig. 4d). The changes in the enzyme production levels were confirmed by zymography analyses of culture supernatants (Additional file 2A–C), which suggested at least four xylanase components were up-regulated in *xlnR* ^A871V^. Notably, the *xlnR* ^A871V^ mutant showed greater increases of cellulase and hemicellulase production than the *gpdA*(p)::*xlnR* strain, particularly in the later phase of cultivation (Fig. 4a–c, e, f; Additional file 3A-C).

When the culture supernatants of *xlnR* ^A871V^ mutant were analyzed by SDS-PAGE, differential protein patterns were clearly observed compared with those in wild-type strain (Fig. 4h). Five of the obviously differential protein bands between the strains were selected and subsequently analyzed by MS–MS (Table 2). The four proteins with higher abundances in *xlnR* ^A871V^ mutant were identified as xylanases (PDE_04478 and PDE_03573) and β-xylosidases (PDE_00049 and PDE_08037), respectively, while the protein with dramatically decreased abundance was found to be glucoamylase Amy15A (PDE_09417). Further qRT-PCR analysis showed that *xlnR* ^A871V^ mutant had 6.9-, 0.9-, 10.6-, and 20.7-fold increases in the transcription of *PDE_00049*, *PDE_03573*, *PDE_04478*, and *PDE_08037*, respectively, compared with those in wild-type strain in cellulose medium (Additional file 4). These data implied that genes encoding xylanases and xylosidases are among the efficiently regulated targets of XlnR^A871V^ in *P*. *oxalicum*.

**Table 2:** Identification of proteins using MS–MS method

Band number	Gene ID	Function prediction	CAZy family	Accession no.	Predicted MW (Da)	Predicted PI	Protein score C.I. %
1	PDE_09417	Glucoamylase Amy15A	GH15	EPS34453	67626.9	5.69	100
2	PDE_03573	Xylanase	GH30	EPS28627	52637.1	5.20	100
3	PDE_00049	β-Xylosidase	GH3	EPS25118	87355.2	5.43	100
4	PDE_04478	Xylanase	GH11	EPS29528	22976.8	6.49	100
5	PDE_08037	β-Xylosidase	GH3	EPS33075	98515.0	4.96	100

### Identification of *PDE_02864*(p) as a strong promoter for manipulation of the cellulase transcriptional regulatory pathway

Previous result showed the dose-controlled regulation of cellulase production by ClrB in *P. oxalicum* [10]. Considering the significant effect of XlnR^A871V^ on the expression of lignocellulolytic enzyme genes, we supposed overexpression of this mutant using promoters stronger than its native promoter would more efficiently improve the enzyme production levels. Four candidate genes including the expansin gene *PDE_02102*, the xylanase gene *PDE_04478*, the 40S ribosomal protein S8 gene *PDE_02864*, and an unannotated gene *PDE_07106* were picked out in consideration of their high transcriptional levels in cellulose medium based on the RNA-Seq results [10] for further exploration of their promoters.

**Fig. 5:**
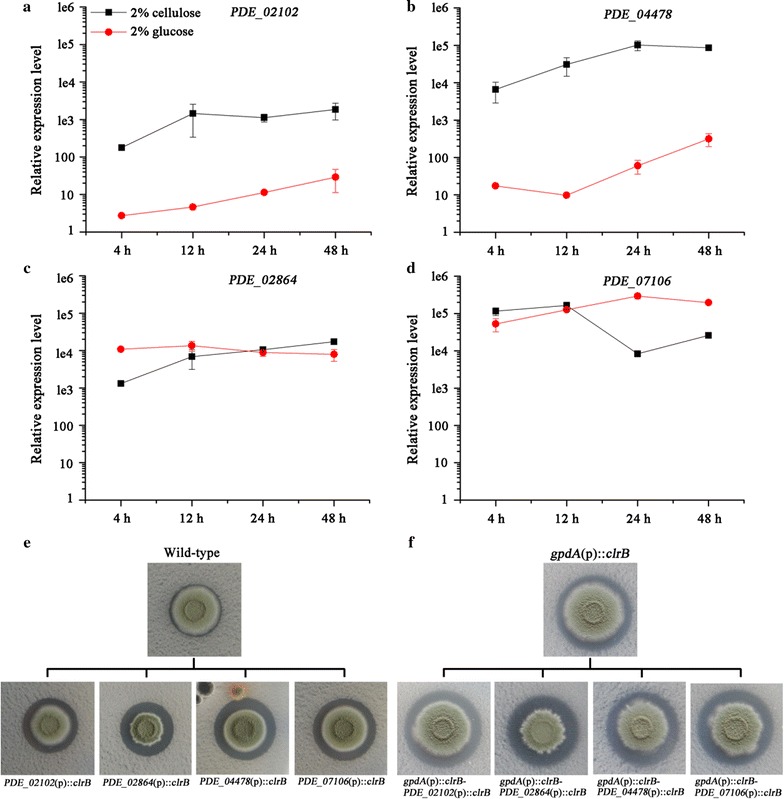
Analysis of the driving efficiencies of four promoters. **a**–**d** Transcriptional levels of four genes in wild-type strain were measured on glucose and cellulose. *Error bars* represent the standard deviations. **e**, **f** Phenotype observations of *clrB* overexpression strains and *clrB* double overexpression strains on cellulose plates

Expressions of the above four genes in wild-type strain were analyzed in the media with glucose or cellulose as sole carbon source. Transcriptional levels of *PDE_02102* and *PDE_04478* showed typical induced expression on cellulose compared with those on glucose (Fig. 5a, b). Transcription of *PDE_02864* showed high and sustained levels under both glucose and cellulose conditions (Fig. 5c). For *PDE_07106*, the transcription decreased at 24 and 48 h on cellulose (Fig. 5d). When the promoters of these four genes were used to drive the overexpression of *clrB*, *PDE_02864*(p)::*clrB* strain showed the greatest increase in the ratio of hydrolysis halo to colony diameter in either wild-type or *gpdA*(p)::*clrB* background [10] (Fig. 5e, f). As expected, constitutive expression of *clrB* was achieved in strains *PDE_02864*(p)::*clrB* and *PDE_07106*(p)::*clrB* (Additional file 5A). Since the FPase activity in *PDE_02864*(p)::*clrB* strain was higher than that in *PDE_07106*(p)::*clrB* strain in wheat bran medium (Additional file 5B), the *PDE_02864* promoter was used for the overexpression of *xlnR* ^A871V^ in the following strain engineering studies.

### Enhancement of lignocellulolytic enzyme production by combining XlnR^A871V^ overexpression with the manipulation of *creA* and *clrB*

**Fig. 6:**
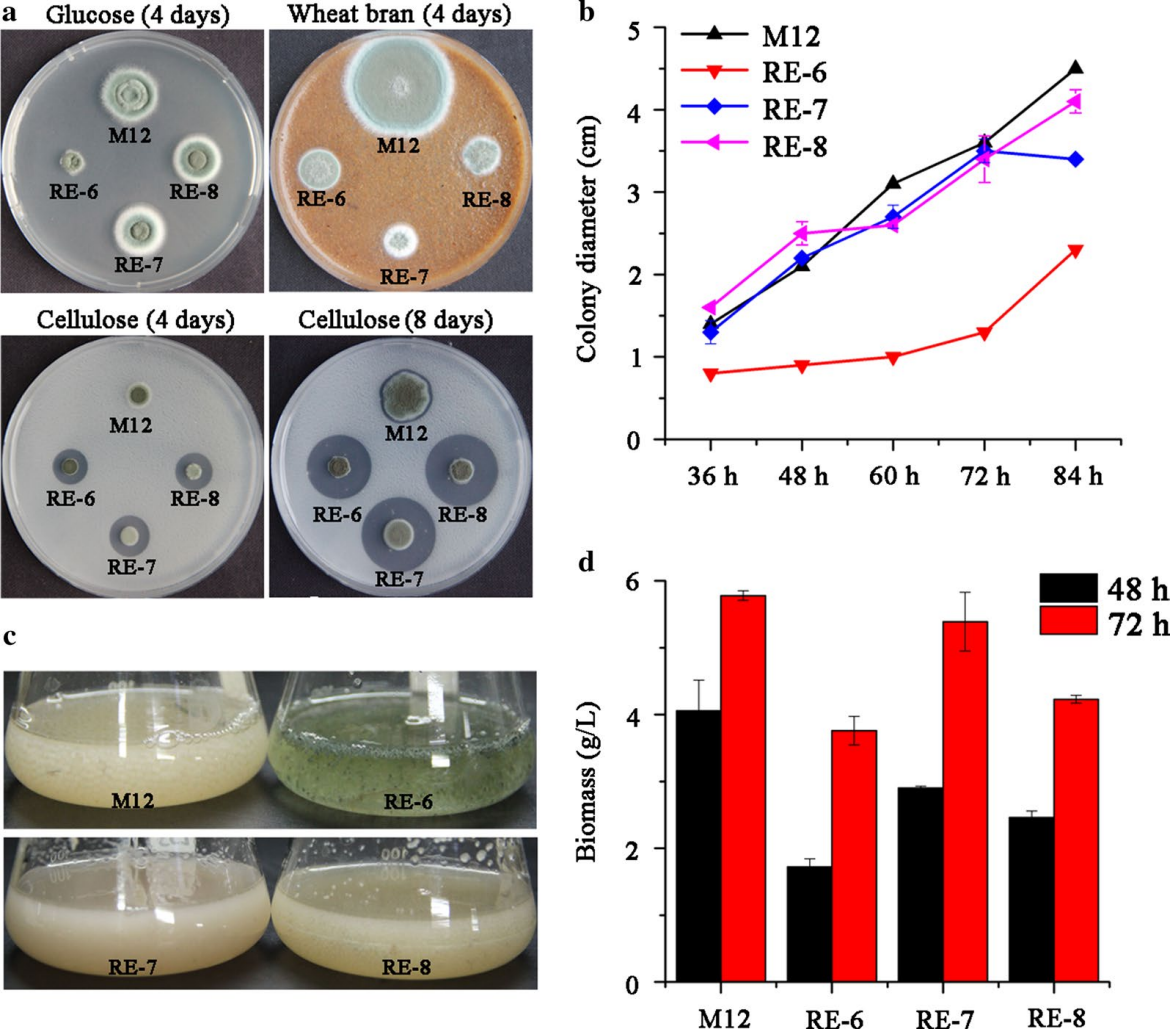
Growth analysis of M12 and its engineered strains. a Phenotype observations of M12 and the engineered strains on different carbon (glucose, wheat bran, and cellulose) plates. **b** Colony size measurements of M12 and the engineered strains on glucose plates. **c** Phenotype observations in liquid glucose medium. **d** Mycelial dry weights of strains grown in glucose medium. *Error bars* represent the standard deviations

**Fig. 7:**
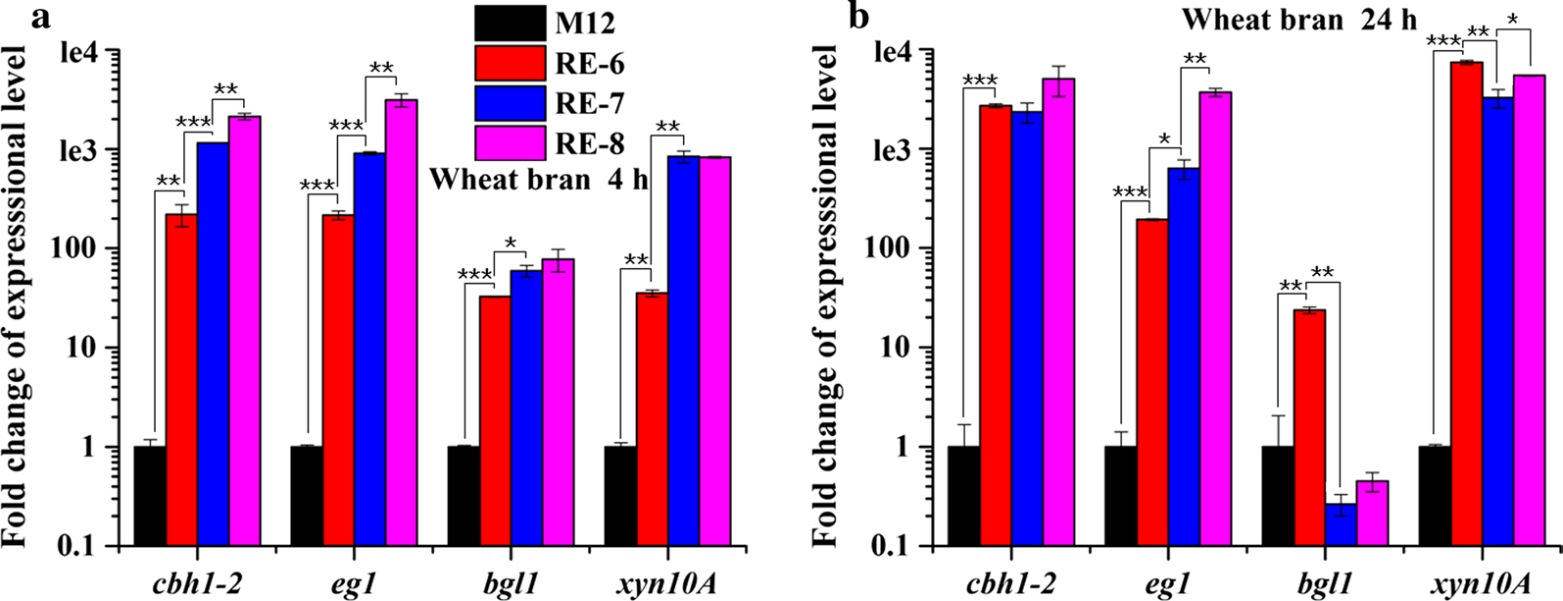
Transcription analysis of lignocellulolytic enzymes in M12 and its engineered strains on wheat bran. Transcriptional levels of lignocellulolytic enzymes in RE-6, RE-7, and RE-8 strains versus those in wild-type strain (set to one) at 4 h (**a**) and 24 h (**b**) were analyzed. *Error bars* represent the standard deviations. Statistical significance of the differences between strains was calculated. *p < 0.05, **p < 0.01, ***p < 0.001

**Fig. 8:**
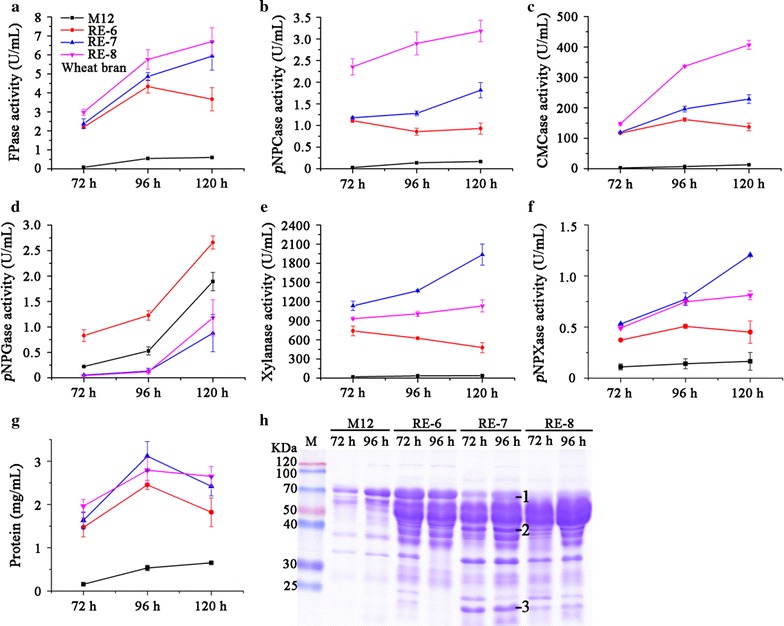
Enzyme activity and extracellular protein analyses of M12 and its engineered strain on wheat bran. FPase (**a**), *p*NPCase (**b**), CMCase (**c**), *p*NPGase (**d**), xylanase (**e**) and *p*NPXase activities (**f**), and extracellular protein concentrations (**g**) of M12, RE-6, RE-7, and RE-8 strains were analyzed. All *error bars* represent the standard deviations. **h** SDS-PAGE analysis of the culture supernatants of M12 and engineered strains

We have previously identified the synergistic effect on improving cellulolytic and xylanolytic gene expressions by overexpression of *clrB* and deletion of *creA* in *P. oxalicum* wild-type strain 114-2 through two-step genetic manipulations [10, 17]. Here, we first validated the efficiency of this combinatorial manipulation in M12 strain [26], a uracil auxotrophic mutant derived from wild-type 114-2. M12 showed similar production of cellulase and xylanase to wild-type strain (Additional file 6A–C). Consequently, a Δ*creA*-*gpdA*(p)::*clrB* cassette with *gpdA*(p)::*clrB* cassette flanked by *creA* upstream and downstream sequences was constructed (Fig. 1b) and transformed into M12. Southern blot (Additional file 1B) and PCR (Fig. 1b) showed one of the transformants contained a single-copy integration of Δ*creA*-*gpdA*(p)::*clrB* cassette at the *creA* gene locus, which was named RE-6 (Table 1) and further characterized. As expected, RE-6 mutant showed increased hydrolysis halo on cellulose plate (Fig. 6a) and significantly enhanced transcription of lignocellulolytic enzyme-encoding genes (Fig. 7a, b) as well as the production of corresponding enzymes (Fig. 8a–f) than those in M12. Notably, increased transcription of *bgl1* (Fig. 7a, b) and production of β-glucosidase (Fig. 8d) were observed in RE-6 compared with those in M12.

Given the synergistic effect of the overexpression of ClrB and XlnR on cellulase expression [10], we assumed the overexpression of *xlnR* ^A871V^ in RE-6 might further enhance cellulase expression. Correspondingly, *PDE_02864*(p)::*xlnR* ^A871V^ cassette (Fig. 1c) was constructed and transformed into RE-6, producing a mutant named RE-7 (Table 1). PCR (Fig. 1c) and Southern blot analyses (Additional file 1C) showed the *PDE_02864*(p)::*xlnR* ^A871V^ expression cassette was integrated into the genome in RE-7. RE-7 showed larger halo on cellulose plate compared with RE-6 (Fig. 6a). In wheat bran medium, RE-7 showed 4.2-, 4.2-, and 23.8-fold increases in transcriptional levels of *cbh1*-*2*, *eg1*, and *xyn10A* compared with those in RE-6 at 4 h (Fig. 7a), respectively. The FPase, *p*NPCase, CMCase, xylanase and *p*NPXase activities, and extracellular protein concentration in RE-7 mutant increased by 0.6-, 1.0-, 0.7-, 3.0-, 1.6-, and 0.3-folds, respectively, when compared with those in RE-6 mutant at 120 h (Fig. 8a–c, e–g). The increases in cellulase and xylanase were also observed in another independent transformant RE-7-2 (Additional file 7A-B). Again, decreased *bgl1* transcription at 24 h (Fig. 7b) and *p*NPGase activity (Fig. 8d) were observed in RE-7 relative to RE-6, which was also revealed by zymography analyses of the culture supernatants (Additional file 2C). Additionally, SDS-PAGE results showed that much more protein bands were detected in RE-7 compared with those in RE-6 mutant (Fig. 8h). Comparison of individual protein bands between strains suggested higher concentrations of xylanases PDE_03573 and PDE_04478 and decreased concentration of amylase Amy 15A in RE-7 mutant than those in RE-6 (Fig. 8h).

### Combinational engineering of cellulolytic transcription factors and main cellulase genes conferred further improved cellulase production

To explore whether engineering of cellulase transcription factors and direct tuning the expression of their target genes could be combined to improve cellulase production, cellulase genes *cbh1*-*2* and *eg1* were chosen for overexpression together with the manipulation of *creA*, *clrB*, and *xlnR*. These two genes *cbh1*-*2* and *eg1* were tightly regulated by ClrB, CreA, and XlnR (Fig. 7) and their products were identified as the major components in the secretome of *P. oxalicum* [10].

In order to construct large DNA fragment containing multi-gene expression cassettes, we used RecET direct cloning technology [26]. The final 14.1-kb fragment obtained contained four independent gene expression cassettes including *PDE_02864*(p)::*xlnR* ^A871V^, *cbh1*-*2*(p)::*cbh1*-*2*, *eg1*(p)::*eg1*, and *pyrG* (as the selection marker), as shown in Fig. 1d. The multi-gene fragment was transformed into RE-6 and generated the RE-8 mutant (Table 1) as verified by PCR (Fig. 1d) and Southern blot analyses (Additional file 1D). RE-8 showed a similar colony on wheat bran but a larger halo on cellulose plate compared with those of parental strain RE-6 (Fig. 6a). As expected, RE-8 displayed 1.8- and 2.2-fold greater *cbh1*-*2* transcriptional levels and 3.5- and 5.9-fold higher *eg1* transcriptional levels than those in RE-7 at 4 h and 24 h, respectively (Fig. 7a, b). In accordance with the mRNA levels, RE-8 mutant showed 0.8- and 0.8-fold increased *p*NPCase and CMCase activities relative to RE-7 (Fig. 8b, c). While the transcriptional level of *xyn10A* was similar between RE-7 and RE-8 (Fig. 7a), xylanase production after 72 h of cultivation was lower in the latter strain (Fig. 8e). The higher level of CMCase production in RE-8 relative to RE-7 was confirmed by zymography analysis (Additional file 2B). Compared with the original strain M12, the quintuple mutant RE-8 showed 10.2-, 18.1-, 30.6-, 29.7-, and 3.9-fold higher FPase, *p*NPCase, CMCase, xylanase, and *p*NPXase activities, respectively (Fig. 8a–c, e–f). Moreover, specific activities of FPase and xylanase (U/mg total protein) in RE-8 at 120 h increased by 1.8- and 6.5-folds, respectively, relative to M12. That is to say, the performance of the enzyme mixture produced by RE-8 might have been enhanced compared with M12 with the same protein loading in lignocelluloses hydrolysis.

### XlnR^A871V^ negatively regulated amylase production

**Fig. 9:**
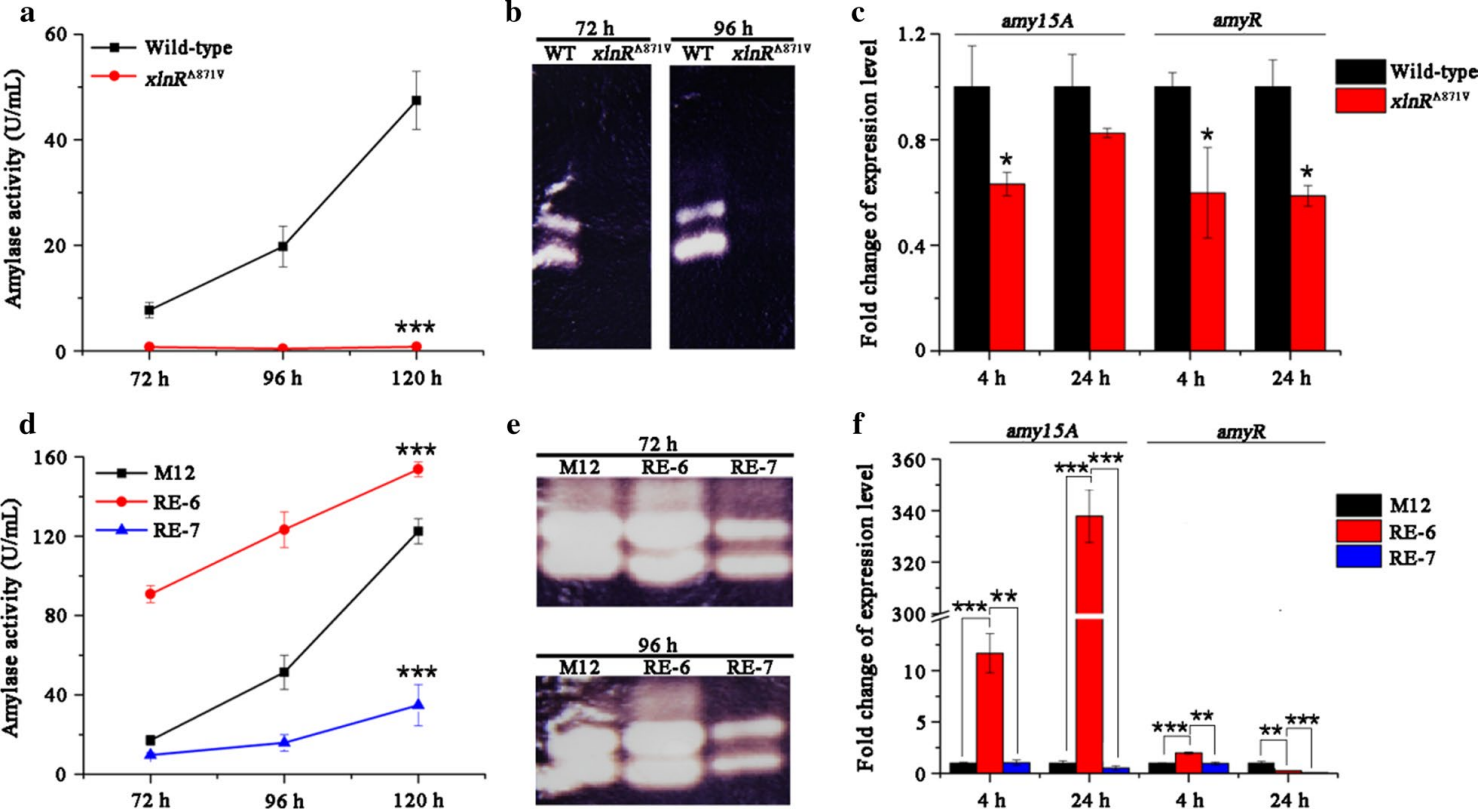
Effect of XlnR^A871V^ on amylase production. **a** Amylase activities of wild-type and *xlnR* ^A871V^ on wheat bran. **b** Zymography analysis of the amylase activities of supernatants of wild-type and *xlnR* ^A871V^ strains. **c** Transcriptional levels of *amy15A* and its regulator *amyR* in *xlnR* ^A871V^ versus those in wild-type strain (set to one). Statistical significance of the differences between wild-type and *xlnR* ^A871V^ at 120 h was calculated. *p < 0.05, **p < 0.01, ***p < 0.001. **d** Amylase activities of M12, RE-6, and RE-7 on wheat bran. **b** Zymography analysis of the amylase activities of supernatants of M12, RE-6, and RE-7 strains.** c** Transcriptional levels of *amy15A* and its regulator *amyR* in RE-6 and RE-7 versus those in M12 strain (set to one).* Error bars* represent the standard deviations. Statistical significance of the differences between strains at 120 h was calculated. *p < 0.05, **p < 0.01, ***p < 0.001

Cellulase and amylase are the major components in the secretome of *P. oxalicum* [11], and the key activator of amylase expression, AmyR, was identified as a negative regulator of cellulase expression [10]. Given the result of SDS-PAGE that the *xlnR* ^A871V^ mutant had a remarkable decrease in the production of the main amylase Amy15A (Fig. 4h), we compared amylase activity and the transcription level of *amy15A* between wild-type and *xlnR* ^A871V^ strains (Fig. 9a–c). Up to 98% decrease in amylase activity was observed at 120 h in *xlnR* ^A871V^ relative to wild-type (Fig. 9a). Consistently, zymography analysis showed that two protein bands with amylase activity were hardly detected in the mutant (Fig. 9b). Besides, the transcriptional levels of *amy15A* and *amyR* in *xlnR* ^A871V^ mutant were about 37 and 18% lower than those in wild-type strain at 4 h and 40 and 41% lower at 24 h, respectively (Fig. 9c). The similar changes were observed by comparing RE-7 carrying *xlnR* ^A871V^ overexpression with its parent strain RE-6 (Fig. 9d–f). While amylase production was enhanced in RE-6 relative to M12 (Fig. 9d, e), further introduction of the *xlnR* ^A871V^ mutation in RE-7 dramatically reduced amylase production (Fig. 9d, e) as well as the transcription of *amy15A* and *amyR* (Fig. 9f) to similar or even lower levels than those in M12.

### Suppression of the phenotype of *creA* deletion by overexpression of XlnR^A871V^

The *cre1* knock-out strain showed smaller colony and reduced hyphal growth in *T. reesei* [27]. Similar phenomenon was also observed in *creA* deletion mutant in *P. oxalicum* [10]. In this study, the colony of RE-6 mutant showed a 50% decrease in diameter than that of M12 strain after 84-h cultivation on glucose plate (Fig. 6b). Interestingly, the growth of RE-7 mutant with the *xlnR* ^A871V^ overexpression showed a recovery on glucose but not on wheat bran (Fig. 6a, b). The recovered growth was also observed in RE-8 (Fig. 6a, b) and other two transformants carrying the XlnR^A871V^ mutation constructed from RE-6 (Additional file 7C). To further investigate this effect of *xlnR* ^A871V^ on growth, conidia of the strains were inoculated in liquid glucose medium and cultivated for 48 h. Similar to the results on glucose plates, the decreased hyphal growth of RE-6 mutant relative to M12 was retrieved with XlnR^A871V^ overexpression (Fig. 6c, d).

## Discussion

In this study, we found that XlnR^A871V^ significantly improved the expression of lignocellulolytic enzyme genes in *P. oxalicum*. The *xlnR* ^A871V^ mutant with a lower *xlnR* level (Fig. 3c, d) had a greater improvement in enzyme production than *gpdA*(p)::*xlnR*, suggesting the high efficiency of engineering the activity but not the abundance of transcription factors. Considering the enhancement of cellulase production by XlnR^A871V^ is less than that of hemicellulase production, combination of XlnR^A871V^ with other regulators of cellulase expression including CreA and ClrB was shown to be efficient for the coordinative production of lignocellulolytic enzymes.

Introduction of XlnR^A871V^ resulted in dramatically reduced amylase production, which could be attributed to the decreased expression of *amy15A* and *amyR*. However, other mechanisms (e.g., changes at protein synthesis and secretion levels) cannot be excluded, as the decrease of *amy15A* expression was less severe than that of amylase activity (Fig. 9a, c). Since AmyR was proved to negatively regulate cellulase expression in cellulose medium in *P. oxalicum* [10], the down-regulation of *amyR* in *xlnR* ^A871V^ mutant could also contribute to the increased cellulase expression. That is to say, XlnR^A871V^ might enhance the expression of lignocellulolytic enzyme genes through both direct and indirect (mediated by AmyR) manners. In addition, the decreased amylase activity in *xlnR* ^A871V^ should release less glucose from starch than wild-type strain in the wheat bran medium, and thus reduced the carbon catabolite repression on lignocellulolytic enzyme expression. Combination of XlnR^A871V^ mutation and deletion of *amyR* is expected to be conducted in the future to elucidate the genetic interaction between the two regulators and may further improve the production of lignocellulolytic enzymes.

One trade-off effect of manipulating the regulatory pathway for improving cellulase production is that the performance of the secreted enzymes could be affected due to changes in the composition of enzyme mixture. For example, introduction of XlnR^A871V^ reduced the production of β-glucosidase (Figs. 4d, 8d), which is a rate-limiting enzyme in the degradation of lignocellulosic materials [25]. Here, the introduction of extra copies of *cbh1*-*2* and *eg1* genes enabled increased production of corresponding enzymes, suggesting the possibility to strengthen the production of specific enzyme components following the engineering of regulatory pathway. Given the fact that efficient degradation of different lignocellulosic materials requires enzyme mixtures with different compositions [28], direct adjustment of the expression of genes encoding individual enzymes should be critically considered in the genetic engineering of strains for the production of optimized enzyme cocktails. It should be pointed out that the overexpression of these enzyme-encoding genes could produce an amplified effect when being performed in strains with engineered regulatory pathways (e.g., RE-6).

Efficient genetic engineering tools, e.g., recycling of selection marker and assembly of large DNA fragments, are required for manipulating multiple genes in the same strain. Here, we used the uracil auxotrophic strain M12 [26] for genetic engineering, where both the presence and absence of selection marker *pyrG* could be easily selected [29]. To ensure *pyrG* being efficiently excised, the *six* sequences recognized by β-recombinase [30, 31] were added outside the *pyrG* cassette in this study (Fig. 1c, d), which allowed further manipulation of other targets using the same marker. In addition, we utilized a RecET protocol in the construction of strain RE-8, which is particularly suitable for fast acquisition of gene cluster containing multiple gene expression cassettes [32].

## Conclusions

In this article, we verified the crucial regulatory function of XlnR^A871V^ on lignocellulolytic enzyme expression in *P. oxalicum*, and combined the manipulation of three regulators (deletion of *creA* and overexpression of *xlnR* ^A871V^ and *clrB*) and two main cellulase genes (overexpressions of *cbh1*-*2* and *eg1*) in one engineered strain. The pentagenic recombinant strain showed significantly improved cellulolytic and hemicellulolytic enzyme production. Our results signify that redesigning the regulation network by combinational manipulation of the activity and abundance of regulators as well as their target genes is a promising strategy for developing lignocellulolytic enzyme hyper-producing strains. Considering the functions of these regulators are conserved in many ascomycete fungi, the strategy should also be suitable to other fungal species.

## Methods

### Strains and culture media

The wild-type strain (CGMCC5302) and all mutants in this article were cultured on wheat bran extract slants to obtain fresh conidia. Vogel’s salts offered the necessary trace elements for hyphal growth in most media unless otherwise noted. Then, 1 L 50× Vogel’s salt solution was prepared. The solution contained 125 g Na_3_Citrate·2H_2_O, 250 g KH_2_PO_4_, 100 g NH_4_NO_3_, 10 g MgSO_4_·7H_2_O, 5 g CaCl_2_·2H_2_O, 0.25 mg biotin, 5 mL trace element solution (5% citric acid·H_2_O, 5% ZnSO_4_·7H_2_O, 1% Fe(NH_4_)_2_(SO_4_)_2_·6H_2_O, 0.25% CuSO_4_·5H_2_O, 0.05% MnSO_4_·H_2_O, 0.05% H_3_BO_3_, and 0.05% Na_2_MoO_4_·2H_2_O, wt/vol), and 755 mL distilled water. The medium for transformation contained 1 M d-sorbitol, 2% glucose, and 1× Vogel’s salt solution. The solid medium for hyphal morphology observation included 1.5% agar and 1× Vogel’s salt solution supplemented with 2% glucose or 1% cellulose, or complex medium referred to simply as wheat bran medium (see below) in this study. The liquid medium containing 2% glucose was used for hyphal growth to produce pre-cultures. The medium containing 2% glucose or 2% cellulose, or the wheat bran medium (2% corn con residue, 0.6% Avicel, 4.66% wheat bran, 1.0% soybean cake powder, 0.2% (NH_4_)_2_SO_4_, 0.28% NaNO_3_, 0.1% urea, 0.3% KH_2_PO_4_, and 0.05% MgSO_4_, w/v), was employed for the mRNA extraction and cellulase enzyme activity analysis. All plates were incubated in a 30 °C incubator, and all liquid cultures were grown in 300 mL flasks at 30 °C and 200 rpm.

### Fungal transformation

Fresh spore solution was added into wheat bran plates and incubated at 30 °C for 14–15 h. The mycelia were collected, and solution S1 (1.2 M d-sorbitol, 0.1 M KH_2_PO_4_, pH 5.6) with 3‰ lysing enzyme from *Trichoderma harzianum* (Sigma-Aldrich, USA) was added to degrade the mycelial cell wall at 30 °C for 3 h. The solution was filtered into a 50 mL centrifuge tube, and the suspension was centrifuged at 2500 rpm and 4 °C for 10 min. The supernatant was discarded, and the sediment was resuspended with 5 mL solution S2 (1 M d-sorbitol, 50 mM CaCl_2_, and 10 mM Tris–HCl at pH 7.5). The suspension was centrifuged at 2500 rpm and 4 °C for 10 min. The supernatant was discarded, and the precipitate was resuspended with 400 μL solution S2 and stored on ice. The transformation system, including 5 μL DNA fragment (concentration ≥ 100 ng/μL), 25 μL solution S3 (25% PEG 6000, 50 mM CaCl_2_, and 10 mM Tris–HCl at pH 7.5), and 100 μL protoplast suspension, was kept on ice for 20 min. Then, 1 mL solution S3 was added to the system, and the system was allowed to stand for another 5 min at room temperature. Afterward, 2 mL solution S2 was added to terminate the reaction. The solution mixture was added into growth medium (1 M d-sorbitol, 2% glucose, 1× Vogel’s salt solution, 0.7% agarose, hygromycin B or pyrithiamine for transformant selection) at 55 °C for plate pouring. After 4-days cultivation at 30 °C, transformants were obtained.

### Construction of *xlnR*^A871V^, RE-6, RE-7, and RE-8 mutants

***xlnR*** ^**A871V**^: The *xlnR* ^A871V^ expression cassette was divided into two parts because of the 871 point mutation. The fragments were, respectively, amplified from the genomic DNA of wild-type strain with primer pairs xmU-F + xmU-R and xmCDS-F + xmCDS-R (Additional file 8). The sequence GCC encoding alanine was replaced by GTC encoding valine in primer xmCDS-F, and GGC was replaced by GAC in primer xmU-R. The *hph* selection maker cassette was amplified from the pSilent-1 plasmid [10] with primer pair hphS-F + hphS-R (Additional file 8). The *xlnR* downstream fragment was amplified with xmD-F + xmD-R (Additional file 8). In accordance with the double-joint PCR method [33], 25 bp homology sequences were, respectively, designed on xmU-R, xmCDS-R, and xmD-F for a four-fragment fusion. Primer pair xmU-F + xmDR was used to amplify the 8.4 kb *xlnR* ^A871V^ cassette. The obtained overexpression cassette was transformed into the wild-type strain.

**RE-6**: The *gpdA*(p)::*clrB* cassette was constructed as previously described [10]. The primer pair gpdA-F2 + ptrA-R1 was used to amplify the *gpdA*(p)::*clrB* cassette in this article. The upstream and downstream fragments of *creA* were amplified with primer pairs creAU-F +creAU-R and creAD-F +creAD-R, respectively. The three fragments were fused in accordance with the double-joint PCR method, and the primer pair creAU-F + creAD-R was used to amplify the final 9.1 kb cassette. The obtained overexpression cassette was transformed into the M12 strain.

**RE-7**: The *xlnR* ^A871V^ native promoter was replaced with the *PDE_02864* promoter. The *PDE_02864* promoter was amplified with primer pair PDE_02864-F + PDE_02864-R. The *xlnR* ^A871V^ open reading frame and 3′ untranslated region were amplified with the primer pair xlnR-F + xlnR-R. The *pyrG* selection marker cassette from *A*. *nidulans* with two six sequences on both sides was amplified with primer pair six-pyrG-F + six-pyrG-R. Three fragments were fused in accordance with the double-joint PCR method. Primer pair PDE_02864-F + six-pyrG-R was used to amplify the 6.7 kb *PDE_02864*(p)::*xlnR* ^A871V^ cassette. The obtained overexpression cassette was transformed into the RE-6 mutant.

**RE-8**: *PDE_02864*(p)::*xlnR* ^A871^, *cbh1*-*2*(p)::*cbh1*-*2*, and *eg1*(p)::*eg1* were each amplified with PDE_02864-F2 + xlnR-R, cbh1-F + cbh1-R, and eg1-F + eg1-R primer pairs. The *pyrG* selection marker cassette from *A*. *nidulans* with six sequences on both sides was amplified with six-pyrG-F2 + six-pyrG-R2 primer pair. The RecET direct cloning method was employed to join the four fragments. The 20 μL ligation system was prepared using the following components: 0.13 μL T4 DNA polymerase, 0.2 μL purified bovine serum albumin 100×, 2 μL NE buffer 2, 5 μL linearized p15A vector [32], and 12.67 μL of all amplified fragments. The system was subjected to the following program: incubation at 25 °C for 30 min, 75 °C for 20 min, and 50 °C for 30 min. The ligation system was spotted on a nitrocellulose membrane (Merck Millipore, Ireland) floating on water for 40 min to remove the metal ions and then stored at −20 °C. *E*. *coli* GBdir-gyrA462 competent cells were prepared and transformed in accordance with modified methods as described by Wang et al. [32]. The obtained overexpression cassette was transformed into the RE-6 mutant.

### Phenotypic observation

Equal conidial suspension (10^7^/mL, 1 μL) was spotted on each plate for phenotype observations. The plates were kept at 30 °C for 4 to 8 days.

### Biomass measurement

Equal spores (10^7^) of strains were inoculated into 100 mL glucose media, and mycelia were gathered at 48 and 72 h. Mycelia were dried at 80 °C for 3 h to constant weight for biomass measurements.

### qRT-PCR

Strains were pre-cultured in 2% glucose medium at 30 °C and 300 rpm for 20 h. Equal amounts of mycelia were transferred to 1× Vogel’s salt solution and cultured for 2 h. Then, the mycelia were transferred to wheat bran/cellulose media. The mycelia were ground in liquid nitrogen and added to 1 mL TRIzol solution. mRNA extraction and cDNA synthesis were strictly performed in accordance with the manufacturer’s instructions of RNAiso™ reagent (TaKaRa, Japan) and PrimeScript RT Reagent Kit (TaKaRa, Japan). The qRT-PCR reaction mixture contained 10 μL SYBR II, 1 μL cDNA template, 1.6 μL primer pair, and 7.4 μL DEPC-treated water. The reaction program was as follows: 95 °C for 2 min, 40 cycles at 95 °C for 10 s, and 61 °C for 30 s. The fluorescence signal was gathered at the end of each extension step at 80 °C, and a melting curve program with the temperature gradient of 0.1 °C/s (from 65 to 95 °C) was performed. The transcriptional level of *actin* (*PDE_01092*) was selected as an internal reference for data normalization. The primers are shown in Additional file 8.

### Southern blot analysis

Three primer pairs (listed in Additional file 8) were designed to amplify probes *xlnR*, *clrB*, and *eg1*, respectively. The genomic DNA of wild-type, *xlnR* ^A871V^, and *gpdA*(p)::*xlnR* strains were, respectively, digested by restriction enzyme *Pst*Ι and hybridized with probe *xlnR* for *xlnR* ^A871V^cassette identification. *Hind*ΙΙΙ was used to digest the genomic DNA of the RE-6 and RE-7 strains for *PDE_02864*(p)::*xlnR* ^A871V^ cassette identification. *Sca*Ι was used to digest the genomic DNA of M12, RE-6, RE-7, and RE-8 for Δ*creA*-*gpdA*(p)::*clrB* cassette identification. *BamH*Ι was used to digest the genomic DNA of RE-6 and RE-8 strains for *PDE_02864*(p)::*xlnR* ^A871V^-*cbh1*-*2*(p)::*cbh1*-*2*-*eg1*(p)::*eg1* cassette identification. DIG-High Prime DNA Labeling and Detection starter Kit I (Roche, Switzerland) was used for probe preparation, fragment hybridization, and immunological detection according to the manufacturer’s instructions.

### Enzyme activity assays

Substrates, including Whatman No. 1 filter paper (Shanghai, China), 10 mg/mL beechwood xylan (Sigma, USA) solution, 1 mg/mL *p*NPC (Sigma, USA) solution, 10 mg/mL CMC-Na solution (Sigma, USA), 1 mg/mL *p*NPG (Sigma, USA) solution, and 10 mg/mL starch (Sigma, USA) solution, were prepared for FPase, xylanase, *p*NPCase, CMCase, *p*NPGase, and amylase activity assays, respectively. For the FPase activity assay, the reaction system, including a 0.05 g Whatman No. 1 filter paper, 1.5 mL acetate buffer (pH 4.8), and 0.5 mL diluted culture supernatant, was maintained at 50 °C for 60 min. Then, 3 mL DNS [10] was added to the reaction system. The reaction system was placed in boiling water for 10 min and 20 mL distilled water was added. Absorbance of the reaction system at 540 nm was determined. Up to 1.5 mL beechwood xylan, CMC-Na, or starch solution was added for xylanase, CMCase, or amylase activity assays, respectively. Reaction at 50 °C for 30 min was offered for xylanase and CMCase activity assays, and reaction at 40 °C for 10 min was offered for amylase activity assay. The concentration of reducing sugars was measured with DNS method. *p*NPCase, *p*NPXase, and *p*NPGase activity assays were as follows: the reaction system including 50 μL *p*NPC/*p*NPG/*p*NPX solution and 100 μL diluted culture supernatant was kept at 50 °C for 30 min, and 150 μL 10% Na_2_CO_3_ was added to stop the reaction. Absorbance of the reaction system at 420 nm was determined. One unit of enzyme activity was defined as the amount of enzyme required to release 1 μmol product (glucose/xylose/*p*-nitrophenyl) from the substrate per minute under the assayed conditions. The concentration of extracellular protein was measured by the Bradford Kit (Sangon, Shanghai, China).

### SDS-PAGE analysis

Up to 12.5% gel was prepared for protein separation. The mixture of 15 μL culture supernatant and 3 μL 5× loading buffer was placed in boiling water for 10 min and was loaded into the gel. Coomassie brilliant blue R250 (Sangon, Shanghai, China) was used to color the gel for 30 min. Destainer (glacial acetic acid:ethanol:distilled water, 1:1:8,v/v/v) was used to destain the gel for another 2–4 h.

### Protein analysis using MS/MS

Protein bands were cut from SDS-PAGE gels and soaked in 30% acetonitrile/100 mM NH_4_HCO_3_ solution for color removal. Then, the supernatant was discarded and protein sample was incubated in 100 mM NH_4_HCO_3_ at room temperature for 15 min. The supernatant was discarded and the samples were freeze-dried with liquid nitrogen. The samples were digested overnight in 12.5 ng/μL trypsin in 25 mM NH_4_HCO_3_ and then extracted thrice with 60% acetonitrile/0.1% TFA. The extracts were pooled and dried completely, and then 2 μL 20% acetonitrile was added to dissolve the extracts and 1 μL sample solution was spotted to the MALDI target and was naturally dried. Up to 0.5 μL 60% acetonitrile/0.1% TFA solution was added and the sample solution was naturally dried. The 5800 MALDI-TOF/TOF (Applied Biosystems, USA) was used to analyze protein peptides. The Nd:YAG laser with 355 nm wave length of exciting light and the 2.2 kV electrospray voltage was applied. MS/MS queries were performed using Mascot search engine 2.2 (Matrix Science, Ltd.). Proteins were identified by searching the *P*. *oxalicum* protein database (http://genome.jgi.doe.gov/Penox1/Penox1.home.html).

### Zymography analysis

Xylan (0.25%), CMC-Na (0.25%), and starch (0.25%) were, respectively, added to the gels for SDS-PAGE to analyze the xylanase, CMCase, and amylase activities of samples. The gels were soaked in 5% Triton X-100 for 1 h to remove SDS. The gels were incubated in sodium acetate buffer (0.2 M, pH 4.8) at 50 °C (40 °C for amylase activity assay) for 30 min for enzymatic reaction. The gel containing xylan or CMC-Na was soaked in the solution containing 0.25% Congo Red with 10% ethanol for 30 min for substrate staining and then the gels were transferred to destaining solution (1 M NaCl) for 20 min. Iodine solution (6% KI and 0.6% I_2_) was used to detect the starch degradation for amylase assay. For β-glucosidase detection, native-PAGE without SDS addition was performed [34], and the gel was soaked in solution including 0.1% aesculin solution and 0.5% FeCl_3_ at 50 °C for 10 min.

### Statistical tests

Statistical tests were done with one-tailed homoscedastic (equal variance) *t* test in Microsoft Excel 20 **.

### Accession numbers

The GenBank accession numbers for the five proteins manipulated in this study are as follows: XlnR, EPS32714; ClrB, EPS31045; CreA, EPS28222; CBH1, EPS32984; EG1, EPS32968.

## Additional files

**Additional file 1.** Fig. S1 Southern bolt analysis of the constructed strains. (A) Probe *xlnR* was used to detect the copy numbers of *xlnR* gene in the wild-type, *xlnR* ^A871V^, and *gpdA*(p)::*xlnR* strains. (B) Probe *clrB* was used to detect the copy numbers of *clrB* in M12, RE-6, RE-7, and RE-8 strains. (C) Probe *xlnR* was used to detect the copy numbers of *xlnR* in RE-6 and RE-7 strains. (D) Probe *eg1* was used to detect the copy numbers of *eg1* in RE-6 and RE-8 strains.

**Additional file 2.** Fig. S2 Activity analysis of lignocellulolytic enzymes by zymography. (A-C) Xylanase, CMCase and β-glucosidase activity analyses of the constructed strains, respectively.

**Additional file 3.** Fig. S3 Enzyme activity analysis of wild-type, *xlnR* ^A871V^ and *gpdA*(p)::*xlnR* strains at 24 h and 48 h. FPase (A), CMCase (B) and xylanase activities (C) of supernatants from wild-type, *xlnR* ^A871V^ and *gpdA*(p)::*xlnR* strains on wheat bran were determined. Error bars represent the standard deviations. Statistical significance of the differences between wild-type and each mutant were calculated. *P < 0.05, **P < 0.01, ***P < 0.001.

**Additional file 4.** Fig. S4 Transcription analysis of xylanase and xylosidase genes in wild-type and *xlnR* ^A871V^ strains. Error bars represent the standard deviations. Statistical significance of the difference between wild-type and each mutant were calculated. *P < 0.05, **P < 0.01, ***P < 0.001.

**Additional file 5.** Fig. S5 Transcription of *clrB* and cellulase activity analyses of *clrB* overexpression mutants. (A) Transcriptional levels of *clrB* in wild-type and four *clrB* overexpression strains on glucose and cellulose. (B) FPase activity analysis of *gpdA*(p)::*clrB*, *PDE_02864*(p)::*clrB* and *PDE_07106*(p)::*clrB* strains on wheat bran. Error bars represent the standard deviations. Statistical significance of the difference between *gpdA*(p)::*clrB* and each of the other two mutants were calculated. *P < 0.05, **P < 0.01, ***P < 0.001.

**Additional file 6.** Fig. S6 Lignocellulolytic enzyme activity analysis of wild-type and M12 strains. FPase (A), *p*NPCase (B) and xylanase activities (C) on wheat bran were determined. Error bars represent the standard deviations.

**Additional file 7.** Fig. S7 Enzyme activity analysis and phenotype observation of the transformant RE-7-2. FPase (A) and xylanase activities (B) of RE-6 and RE-7-2 strains on wheat bran at 72 h, 96 h and 120 h were determined. Error bars represent the standard deviations. (C) Phenotypic analysis of RE-6 and three parallel transformants (RE-7, RE-7-1 and RE-7-2) on glucose plate after 4 days’ cultivation.

**Additional file 8.** Table S1 Primers used in this study.

## Abbreviations

CBH: Cellobiohydrolase
EG: Endoglucanase
BGL: β-Glucosidase
CMC: Carboxymethyl cellulose
FPase: Filter paper enzyme
*p*NPC: 4-Nitrophenyl-β-d-cellobioside
*p*NPG: 4-Nitrophenyl-β-d-glucopyranoside
*p*NPX: 4-Nitrophenyl-β-d-xyloside
SDS-PAGE: Sodium dodecyl sulfate-polyacrylamide gel electrophoresis
