# Supplementary figures and images for: Combining manipulation of transcription factors and overexpression of the target genes to enhance lignocellulolytic enzyme production in Penicillium oxalicum
Source: Biotechnol Biofuels. 2017 Apr 20;10:100. doi: 10.1186/s13068-017-0783-3 (PMC5397729; doi:10.1186/s13068-017-0783-3)

**Fig. S1**


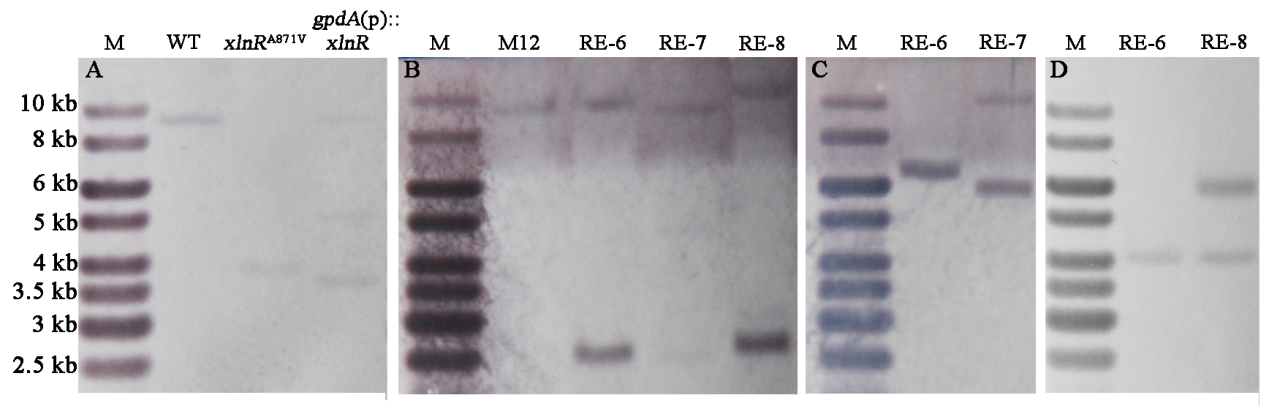

Supplement: Supplementary file 1 — Additional file 1. Fig. S1 Southern bolt analysis of the constructed strains. (A) Probe xlnR was used to detect the copy numbers of xlnR gene in the wild-type, xlnR A871V, and gpdA(p)::xlnR strains. (B) Probe clrB was used to detect the copy numbers of clrB in M12, RE-6, RE-7, and RE-8 strains. (C) Probe xlnR was used to detect the copy numbers of xlnR in RE-6 and RE-7 strains. (D) Probe eg1 was used to detect the copy numbers of eg1 in RE-6 and RE-8 strains. [file 13068_2017_783_MOESM1_ESM.docx]

**Fig. S2**


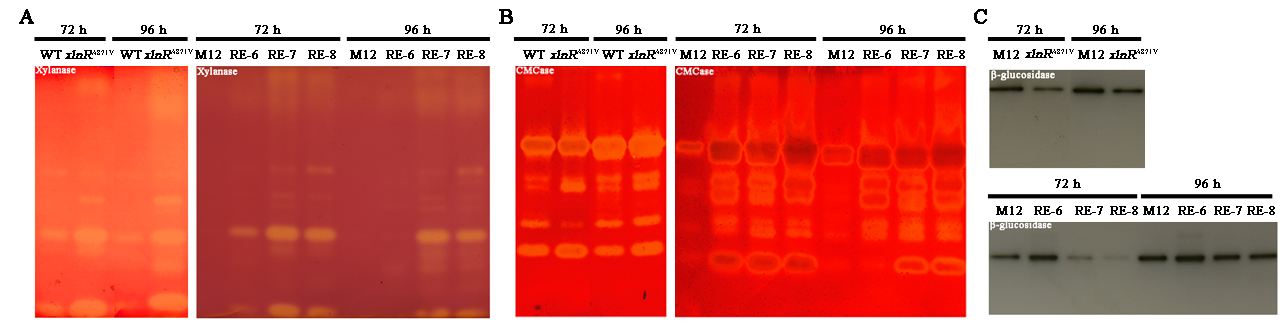

Supplement: Supplementary file 2 — Additional file 2. Fig. S2 Activity analysis of lignocellulolytic enzymes by zymography. (A-C) Xylanase, CMCase and β-glucosidase activity analyses of the constructed strains, respectively. [file 13068_2017_783_MOESM2_ESM.docx]

**Fig. S3**


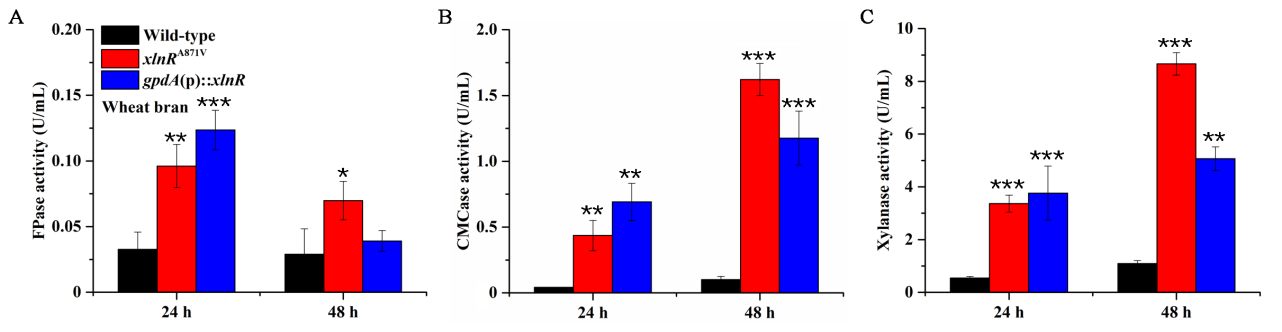

Supplement: Supplementary file 3 — Additional file 3. Fig. S3 Enzyme activity analysis of wild-type, xlnR A871V and gpdA(p)::xlnR strains at 24 h and 48 h. FPase (A), CMCase (B) and xylanase activities (C) of supernatants from wild-type, xlnR A871V and gpdA(p)::xlnR strains on wheat bran were determined. Error bars represent the standard deviations. Statistical significance of the differences between wild-type and each mutant were calculated. *P < 0.05, **P < 0.01, ***P < 0.001. [file 13068_2017_783_MOESM3_ESM.docx]

**Fig. S4**


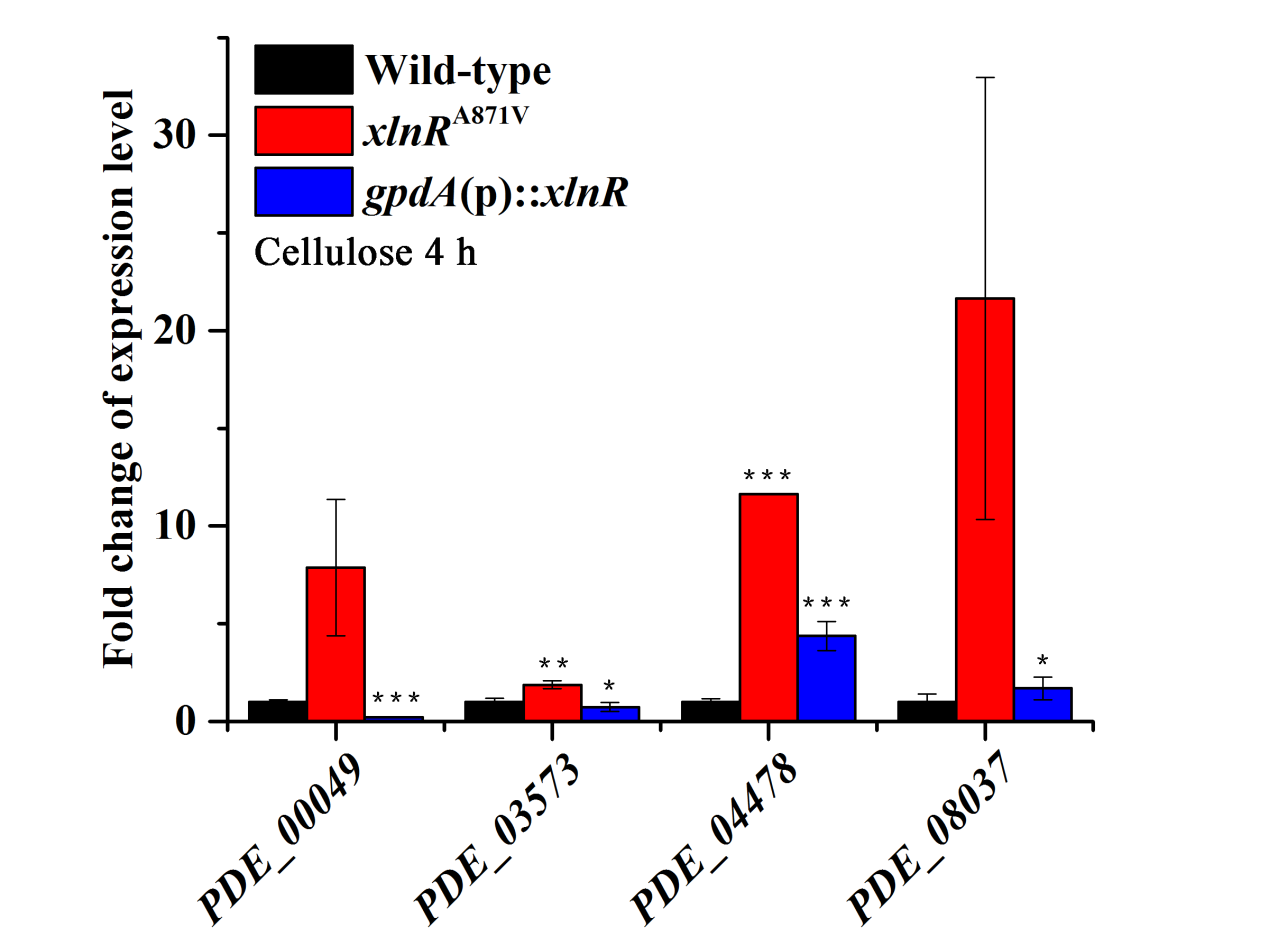

Supplement: Supplementary file 4 — Additional file 4. Fig. S4 Transcription analysis of xylanase and xylosidase genes in wild-type and xlnR A871V strains. Error bars represent the standard deviations. Statistical significance of the difference between wild-type and each mutant were calculated. *P < 0.05, **P < 0.01, ***P < 0.001. [file 13068_2017_783_MOESM4_ESM.docx]

**Fig. S5**


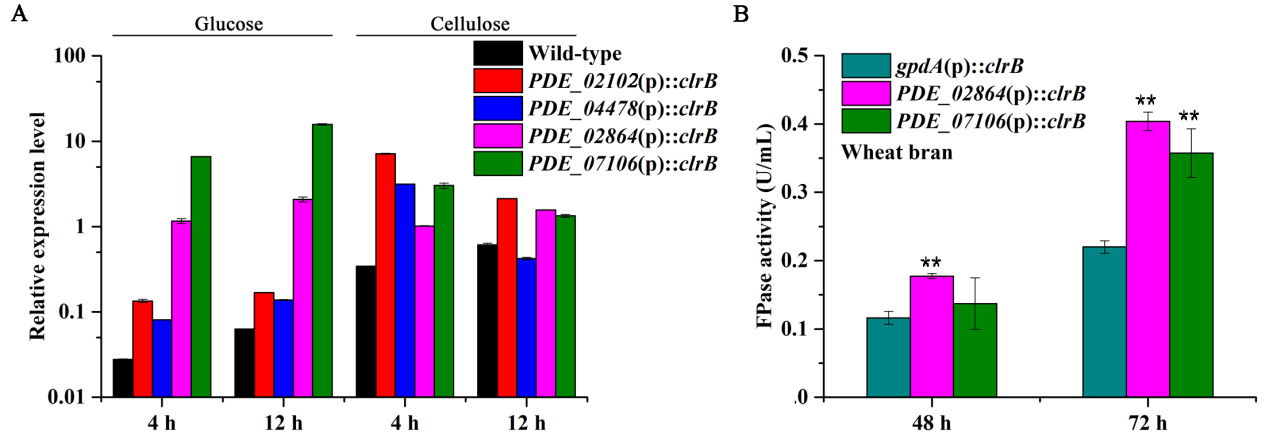

Supplement: Supplementary file 5 — Additional file 5. Fig. S5 Transcription of clrB and cellulase activity analyses of clrB overexpression mutants. (A) Transcriptional levels of clrB in wild-type and four clrB overexpression strains on glucose and cellulose. (B) FPase activity analysis of gpdA(p)::clrB, PDE_02864(p)::clrB and PDE_07106(p)::clrB strains on wheat bran. Error bars represent the standard deviations. Statistical significance of the difference between gpdA(p)::clrB and each of the other two mutants were calculated. *P < 0.05, **P < 0.01, ***P < 0.001. [file 13068_2017_783_MOESM5_ESM.docx]

**Fig. S6**


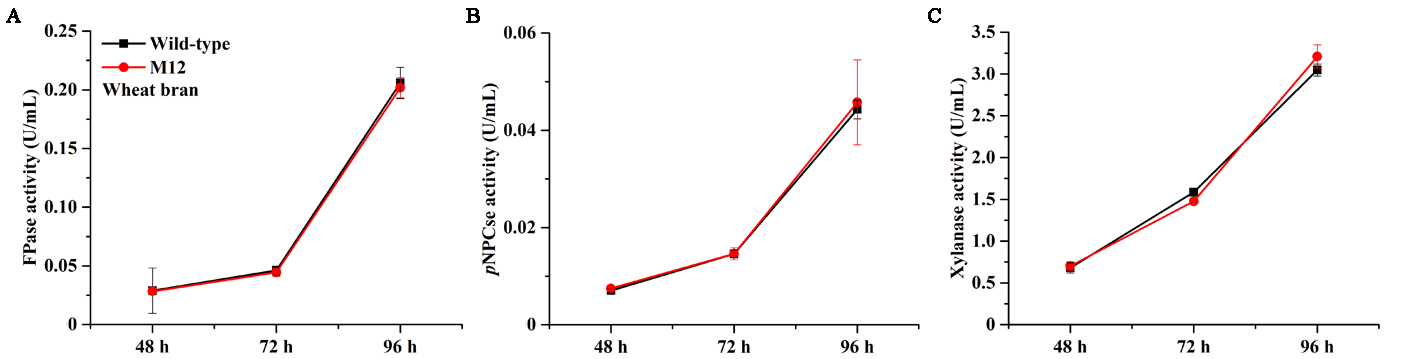

Supplement: Supplementary file 6 — Additional file 6. Fig. S6 Lignocellulolytic enzyme activity analysis of wild-type and M12 strains. FPase (A), pNPCase (B) and xylanase activities (C) on wheat bran were determined. Error bars represent the standard deviations. [file 13068_2017_783_MOESM6_ESM.docx]

**Fig. S7**


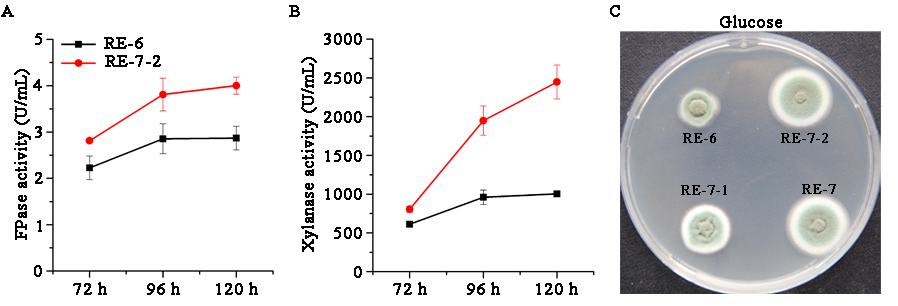

Supplement: Supplementary file 7 — Additional file 7. Fig. S7 Enzyme activity analysis and phenotype observation of the transformant RE-7-2. FPase (A) and xylanase activities (B) of RE-6 and RE-7-2 strains on wheat bran at 72 h, 96 h and 120 h were determined. Error bars represent the standard deviations. (C) Phenotypic analysis of RE-6 and three parallel transformants (RE-7, RE-7-1 and RE-7-2) on glucose plate after 4 days’ cultivation. [file 13068_2017_783_MOESM7_ESM.docx]
